# Impact of COVID-19 Lockdown on Oxidative Potential of Particulate Matter: Case of Athens (Greece)

**DOI:** 10.3390/toxics10060280

**Published:** 2022-05-25

**Authors:** Despina Paraskevopoulou, Aikaterini Bougiatioti, Pavlos Zarmpas, Maria Tsagkaraki, Athanasios Nenes, Nikolaos Mihalopoulos

**Affiliations:** 1 Institute for Environmental Research and Sustainable Development, National Observatory of Athens, 15236 Athens, Greece; nmihalo@noa.gr; 2 Environmental Chemical Processes Laboratory, Department of Chemistry, University of Crete, 71003 Heraklion, Greece; pzarmpas@gmail.com (P.Z.); tsagaraki-maria@hotmail.com (M.T.); 3 LAPI, ENAC, École Polytechnique Fédérale de Lausanne, CH-1015 Lausanne, Switzerland; athanasios.nenes@epfl.ch; 4 Institute of Chemical Engineering Sciences, Foundation for Research and Technology—Hellas, 26504 Patras, Greece

**Keywords:** ROS, DTT, oxidative potential, aerosol toxicity, COVID, fine particulate matter

## Abstract

This work evaluates the aerosol oxidative potential (OP) and its changes from modified air pollution emissions during the COVID-19 lockdown period in 2020, with the intent of elucidating the contribution of aerosol sources and related components to aerosol OP. For this, daily particulate matter (PM) samples at an urban background site were collected and analyzed with a chemical (acellular) assay based on Dithiothreitol (DTT) during the COVID-19 restriction period in Athens (Greece). The obtained time-series of OP, PM_2.5_, organic matter (OM) and SO_4_^2−^ of the pre-, post- and lockdown periods were also compared to the data of the same time periods during the years 2017–2019. Even though all traffic-related emissions have been significantly reduced during the lockdown period (by 30%), there is no reduction in water-soluble OP, organics and sulfate concentrations of aerosol during 2020. The results reveal that the decrease in traffic was not sufficient to drive any measurable change on OP, suggesting that other sources—such as biomass burning and secondary aerosol from long-range transport, which remained unchanged during the COVID lockdown—are the main contributors to OP in Athens, Greece.

## 1. Introduction

Exposure to particulate matter (PM) has long been associated with adverse health outcomes, although over the last few years aerosol toxicity metrics are emerging as better predictors of health risk factors [1,2,3]. Toxicological studies have shown that PM exposure can induce oxidative stress in humans [4]. The latter is attributed to the imbalance between the natural antioxidant capability and the generation of reactive oxygen species (ROS), which is aggravated at a cellular level upon exposure to PM. As a consequence, the term oxidative potential (OP) represents the ability of airborne pollutants to induce oxidative stress, through the production of redox active species that oxidize specific molecules.

Recent findings recognized that the composition, in addition to the mass concentration of PM, plays an important role on the aerosol OP; since the redox active aerosol components present small contributions to the total particulate matter mass [5]. Studies have shown that metals, polycyclic aromatic hydrocarbons (PAHs) and quinones present important toxicity; while PM components like ions could be less toxic [6]. The selected studied fine fraction of particulate matter (PM_2.5_; aerodynamic diameter < 2.5 μm) adsorbs pollutants that are strongly associated with the biological activities and toxicity (poisonous components, such as heavy metals, PAHs, and volatile organic compounds—VOCs), while it reaches deeper into the human lungs due to its aerodynamic diameter. Additionally, PM_2.5_ appears to transcend the annual average concentration limit (10 μg m^−3^) in most of the inhabited regions worldwide [7], and has been proven to be a critical mortality parameter [7] linked to diseases such as lung cancer [8], vascular diseases [9], chronic obstructive pulmonary disease [10], and asthma [11].

The toxicological impact of the aerosol to oxidative stress has been studied using several cellular and acellular methods [12,13], in an attempt to estimate the oxidative and inflammatory activity. The cell-based assays include dichlorofluorescein (DCF), dihydroethidium (DHE), ferricytochrome-c and 4,5-dimethylthiazol-2-yl-2,5-diphenylte-trazolium bromide (MTT), that may significantly demonstrate the cell damage caused by exposure to particles; nevertheless, it cannot provide insights on the mechanism that causes damage, such as oxidative stress [14] and references therein). The chemical (acellular) assays include dithiothreitol (DTT) and ascorbic acid (AA), which can be performed without strictly controlled conditions and allow an instant estimation of the aerosol OP [15,16]. In the current study, the DTT assay method was selected, since it is highly preferred in the acellular OP studies due to its sensitivity to transition metals and organic species [17,18,19]. More specifically, the DTT assay method appears to reflect the in vivo depletion rate of antioxidants caused by the aerosol components [20]. As described in detail by Fang et al. [21] and Paraskevopoulou et al. [22], a two-step reaction takes place during the applied method; the consumption rate of DTT is the indirect measure of the capability of redox active species in PM to catalytically transfer electrons from DTT to oxygen (O_2_). DTT (a chemical surrogate to cellular reductants, such as NADH or NADPH) reduces O_2_ to superoxide anion (O_2_^−^) [23].

The location of the studied site in the Eastern Mediterranean is of particular interest, due to the long-range aerosol transport of airmasses from the Sahara, Asia and Northern Europe [24,25,26,27], as well as strong local aerosol emissions driven by intense vehicular traffic and biomass burning processes for heating purposes [28,29,30]. The coldest period of the year is characterized by the dominance of wood burning for domestic heating, resulting in intense smog events; while, during the warmest period of the year, long-range transportation and secondary aerosol formation processes appear to contribute significantly [31,32,33].

The current research constitutes a case study that took place during the quarantine and lockdown measures that were implemented to limit the COVID-19 pandemic spread (caused by the pneumonia coronavirus SARS-CoV-2) in the region of Greece. The government, similarly to other countries worldwide, decided in March 2020 to apply confinement measures, to reduce the transmission of COVID-19, resulting in restriction of most human activities. The latter limitation of activities (with the exception of essential industries and services) led to critical impacts on economic activities; but on the other hand, the air quality appeared to improve around the world, as an effect of the decreasing atmospheric pollutant emissions ([8] and references therein).

This study reclaims an unprecedented experimental case to estimate the impact of significantly reduced anthropogenic emissions (such as vehicular traffic—among others) on the OP of atmospheric particulate matter. To our knowledge, there is only one study reported on aerosol toxicity during these scarce COVID-lockdown atmospheric conditions, which was performed in China but does not provide a clear indication on the variation of the levels of aerosol OP during the lockdown period, due to a limited number of samples as stated by the authors [34]. Additionally, at the studied site there is a rare European long-term data series of fine aerosol OP (since July 2016) that allows us to study the interannual variability between the OP levels during the COVID lockdown period and the corresponding time intervals during the three previous years (2017–2019). This research on particulate matter toxicity, can contribute significantly to the typification of the redox active aerosol components, contributing to the enactment of environmental policies that lead to healthier living environments.

## 2. Materials and Methods

### 2.1. Sampling Periods

The Government in Greece decided to implement mobility restrictions, starting on 27 February, as described in detail by Grivas et al. [35]. Briefly, the measures were continuously augmented until 13 March, when a general lockdown was imposed at a national level. This resulted in significant reduction in vehicles’ circulation, since the inhabitants could only move for work, health or essential supply purposes and moving for any other reason was prohibited within and outside the municipal areas. The refutation of the measures gradually began on the 4 May, and on 11 May activities like free circulation of passenger vehicles and inhabitants returning to work and commerce operations, were in force.

As a consequence, the current investigation of the aerosol parameters (Oxidative Potential, PM_2.5_ mass concentrations, OM and sulfates), is applied to three different time intervals of the year 2020, as also in Grivas et al. [35]. The pre-lockdown period (PreL) from 1–22 of March, the lockdown period (LocD) from 23 March to 10 May and, finally, the post-lockdown period (PostL) from 11 to 31 of May.

Additionally, the studied parameters of the aforementioned three time periods of 2020, were compared with those of the previous three years at the current site (2017–2019) to make accurate conclusions on the impact of lockdown, as measured using OP levels. The statistically significant differences between all the compared datasets were tested through application of the non-parametric Mann–Whitney test.

### 2.2. Sampling

**Figure 1:**
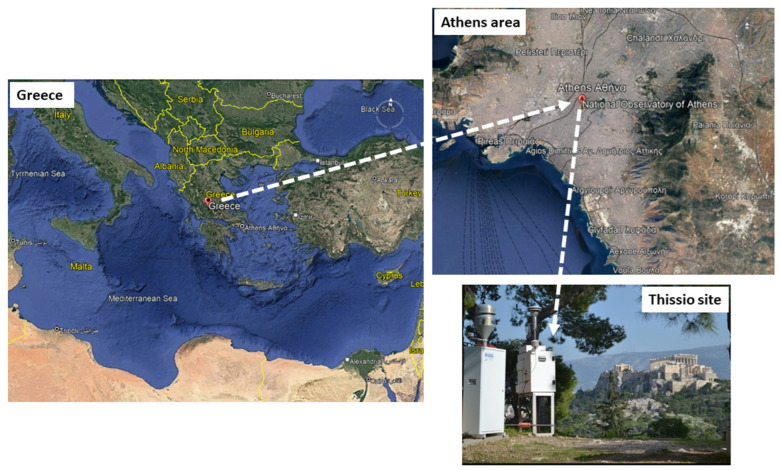
Satellite image of Greece, zooming into the city center of Athens, and focusing on the urban background site of Thissio at the top of Nymphs Hill in central Athens (37.97326° N, 23.71836° E, 105 m a.s.l).

Fine particulate matter was collected at the site of Thissio, within the premises of the National Observatory of Athens (http://apcg.meteo.noa.gr/index.php/infrastructure/thissio-ams, accessed on 16 May 2022). It consists of an urban background station since it is surrounded by pedestrian zones, while it is located at the center of Athens (Figure 1), reflecting the urban environment of the city. During the last years, the site has been described in detail by several research studies, such as Paraskevopoulou et al. [30], Gratsea et al. [36], Liakakou et al. [37] and Panopoulou et al. [38]. For the whole studied period, the daily aerosol fraction of PM_2.5_ (diameter < 2.5 μm) was collected on quartz fiber filters (47 mm, PALLFLEX), using a Low-Volume Sampler (Derenda Sampler LVS 3.1—PNS 15), on a 24 h basis (6 pm to 6 pm LT)—with the exception of year 2019 when 24 h samples were collected every two/three days covering both weekdays and weekends. As mentioned above, the current study includes sampling periods from March to May, during the four consecutive years of 2017 to 2020. Samples were collected and stored in a freezer (~80 °C) until analysis. Filter blanks and field blanks were also prepared and utilized.

### 2.3. PM_2.5_ Mass

Mass concentrations of the PM_2.5_ samples were determined gravimetrically using the US EPA RFPS-1298–126 method, as described in detail by Paraskevopoulou et al. [32], and a Mettler Toledo MX5 microbalance. Briefly, all samples were conditioned (RH: 40 ± 5%, T: 20 ± 3 °C) for 2 days before each weighing. Filter blanks and field blanks were handled accordingly.

### 2.4. Ion Concentration

The main ionic species’ concentrations were measured using a Dionex-500 ion chromatograph. For the determination of the anions’ concentration (Cl^−^, Br^−^, NO^3−^, SO_4_^2−^, PO_4_^3−^, C_2_O_4_^2−^) an Ion Pac AS4A-SC column and an AG4A-SC pre-column, with an ASRS-300 suppressor was used, as described in detail by Paraskevopoulou et al. [32]. The analysis took place through isocratic elution of 20 mM MSA (methanesulphonic acid) at a flow rate of 1.0 mL min^−1^, while punches of the collected quartz filters were analyzed after extraction with nanopure water in an ultrasonication bath and filtration with syringe filters. The detection limit for anions was 20 ppb and, all reported concentrations were blank corrected.

### 2.5. Organic and Elemental Carbon

Organic (OC) and elemental carbon (EC) concentrations were calculated through a thermal optical transmission technique, by a Sunset Laboratory (Oregon) carbon analyzer. Punches of 1 cm^2^ from all quartz filters (samples and blanks) were analyzed based on the method described in detail by Paraskevopoulou et al. [31], using the EUSAAR-2 protocol [39]. The detection limits for OC and EC were 0.26 and 0.05 μg C cm^−2^, respectively, and all the results were blank corrected. Conversion of OC to OM (organic matter) was performed by applying a factor of 1.6, which has been estimated through the online high resolution Aerosol Chemical Speciation Monitor (ACSM) measurements established at our site [33], and is considered indicative of urban environments [40,41].

### 2.6. Oxidative Potential (OP)

The determination of the aerosol water soluble OP took place using the semi-automated system and the dithiothreitol (DTT) assay method described in detail by Paraskevopoulou et al. [22]. Briefly, punches of all collected samples were extracted with nanopure water in an ultrasonication bath, following filtration of the extract with syringe filters prior to the analysis (coefficient of variation, CV = 4%). During this determination, the components of PM oxidize the DTT, in accordance with the reactions and conditions provided by Fang et al. [21]. In short, it consists of a two-step reaction, where the DTT determination step (second step) is taking place at a specified time interval of 4 min. In the final stage of the process, the light absorbing 2-nitro-5-thiobenzoic acid (TNB) is utilized for the determination of the DTT consumption rate, which is expressed as nmol/min/m^3^ (volume normalized DTT activity—DTTv), taking into consideration the sampling air flow of the analyzed PM_2.5_ samples [21], to be comparable with all combined studied aerosol parameters expressed as μg m^−3^. Finally, for the precision control of the semi-automated system, a phenanthrequinone (PQN) solution was used as an external standard, for each analyzed sequence (a total of 12 aerosol samples in every sequence). All reported data were blank corrected.

## 3. Results and Discussion

The meteorological parameters at the studied site, between the PreL, LocD and PostL periods as well as the previous years (2016–2020), have been thoroughly investigated by Grivas et al. [35]. It has been demonstrated that the meteorological conditions occurred during the 2020 study periods were similar to those of the previous four years, indicating that any observed variation of the investigated parameters during the lockdown period was not affected by meteorology, but was mostly driven by changes in the emissions of atmospheric pollutants.

### 3.1. Changes of Measured Parameters during the COVID-19 Lockdown Period

#### Oxidative Potential

**Figure 2:**
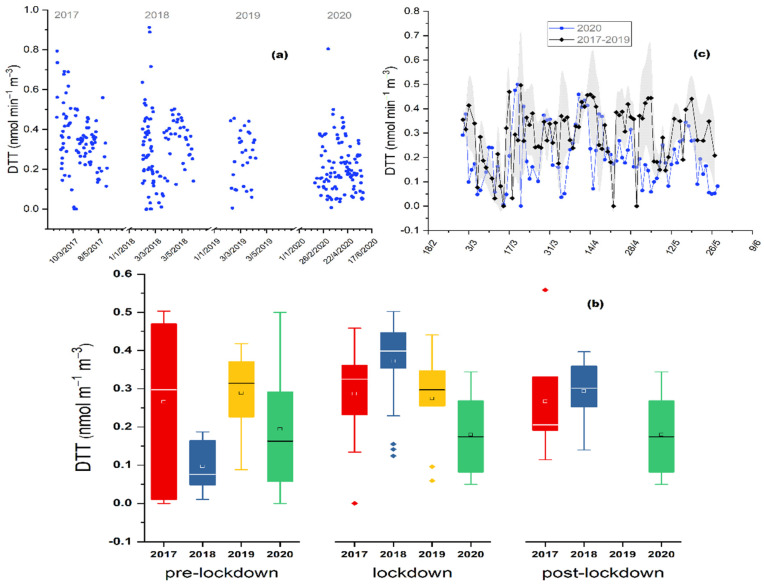
(**a**) Values of water-soluble DTT activity (nmol min^−1^ m^−3^), from March 1 to May 10, for the years 2017 to 2020; (**b**) Average values and standard deviations of water-soluble DTT activity (nmol m^−1^ m^−3^); for pre-lockdown (1–22 March), lockdown (23 March–10 May) and post-lockdown period, from 2017 to 2020, at the Thissio site. The dots represent outlier values; (**c**) Water-soluble DTT activity (nmol m^−1^ m^−3^) 3-year (2017–2019) average daily values (standard deviations marked as grey area), compared to daily values of 2020, from 1 March to 10 May, at the Thissio site.

During the three studied periods of 2020 (PreL, LocD, PostL), the mean aerosol OP values were calculated on a 24-h basis, giving an average of 0.19 ± 0.16, 0.22 ± 0.11 and 0.18 ± 0.10 nmol min^−1^ m^−3^, for PreL, LocD and PostL period, respectively (Figure 2b). This comparison revealed that the DTTv activity during the LocD period appears to slightly increased (by 12%, *p* = 0.30, and 21% *p* = 0.31) when compared with the PreL and the PostL period, respectively, which does not constitute a statistically significant difference, as indicated by the applied Mann–Whitney test. A comparison of the estimated DTTv activity with other areas around the world is not feasible, due to absence of OP data.

OP levels were not reduced during the lockdown period, even though the emission of most primary air pollutants, especially those related to traffic, such as NO_2_, CO and CO_2_, appeared to have been significantly reduced at the studied area by almost 40% (*p* < 0.05) [35]. Overall, the current study highlights that aerosol sources like vehicular traffic could possess a small contribution to the OP of fine particulate matter, since traffic-related pollutants were reduced significantly during the LocD period at Thissio site [34], which is consistent with other reported urban environments upon lockdown implementation [42,43,44,45,46,47]. During the lockdown, neither biomass burning, nor long-range transport was reduced; this could be a plausible explanation why no significant changes were observed for OP levels.

A previous OP study at our site [22] demonstrated that there are distinct aerosol sources affecting the fine mode OP during wintertime. Biomass burning was identified as a major source while, BC, organics and SO_4_^2−^ were highly associated with OP. Overall, combustion processes and long-range transport of aerosol contributed significantly to the toxicity of aerosol on a seasonal basis. As a consequence, the aerosol mass concentration, sulfates, organic matter, and BC concentrations influencing OP levels were also examined during the three periods.

### 3.2. PM_2.5_ Mass Concentrations

**Figure 3:**
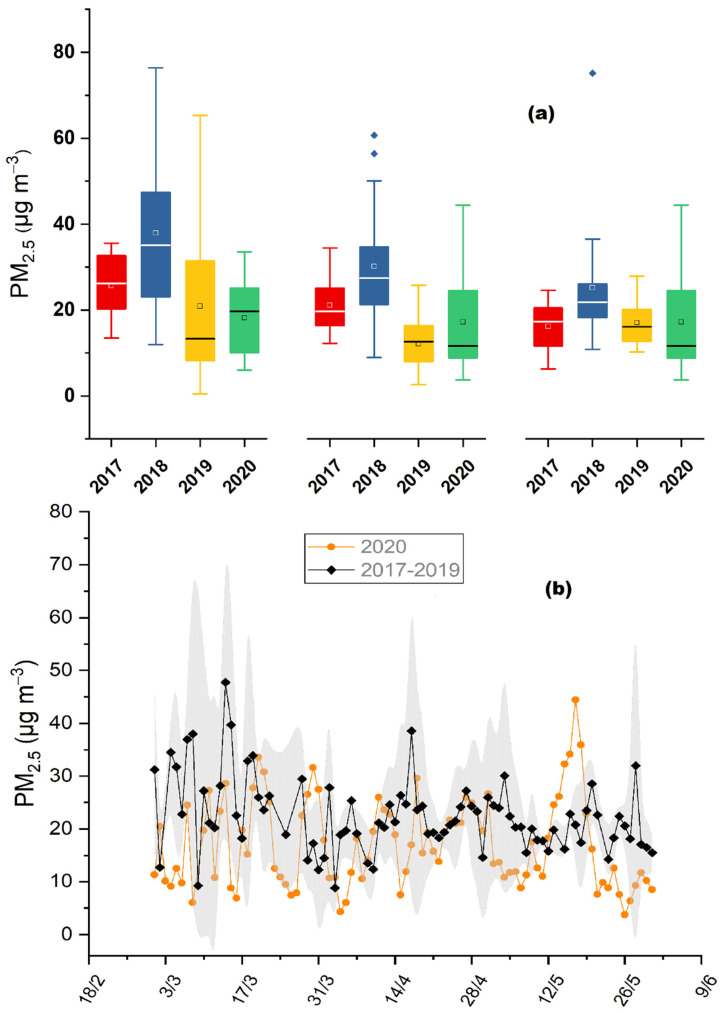
(**a**) Average values and standard deviations of PM_2.5_ mass concentrations (μg m^−3^); for pre-lockdown (1–22 March), lockdown (23 March–10 May) and post-lockdown period, from 2017 to 2020, at the Thissio site. The dots represent outlier values; (**b**) PM_2.5_ mass concentrations (μg m^−3^) average of 3-year (2017–2019) daily values (standard deviations marked as grey area), compared to daily values of 2020, from 1 March to 10 May, at the Thissio site.

The daily mass concentrations of PM_2.5_ measured for the studied periods of PreL, LocD and PostL (Figure 2b), were found to be at 18.16 ±8.75, 16.67 ±6.78 and 17.23 ±11.54 μg m^−3^, respectively (Figure 3a). The results reveal a decrease in ambient aerosol masses through the lockdown period (at a percentage of 8%, *p* = 0.72, when compared to the pre-lockdown and of 3%, *p* = 0.21, regarding the post-lockdown period), which coincides with additional studies worldwide reporting that changes in PM were less pronounced compared to the primary pollutants [48,49]. The observed decline could be linked to the accelerated reduction in air pollutants during the lockdown; while its small percentage demonstrates the complex formation mechanisms of fine particulate matter that include mostly secondary and long—range transportation processes, and to a lesser extent primary emissions. During the lockdown period, activities like vehicular circulation were significantly reduced (about 40–50%, compared to the same time period in 2019–according to the Greek Ministry of Infrastructure and Transport)–but on the other hand, activities like energy consumption, which also contribute notably to the PM_2.5_ formation, presented minimal reduction, providing a more explicit justification for the observed variation of fine aerosol mass concentrations. Our result for the PM mass decrease was in excellent agreement with [50], who performed comparative analysis of PM_2.5_ between the 50 most populated countries under lockdown conditions, in the European cities and reported a reduction in PM_2.5_ mass concentration as low as 5%.

### 3.3. Sulfate, Organic Matter and Black Carbon Concentrations

**Figure 4:**
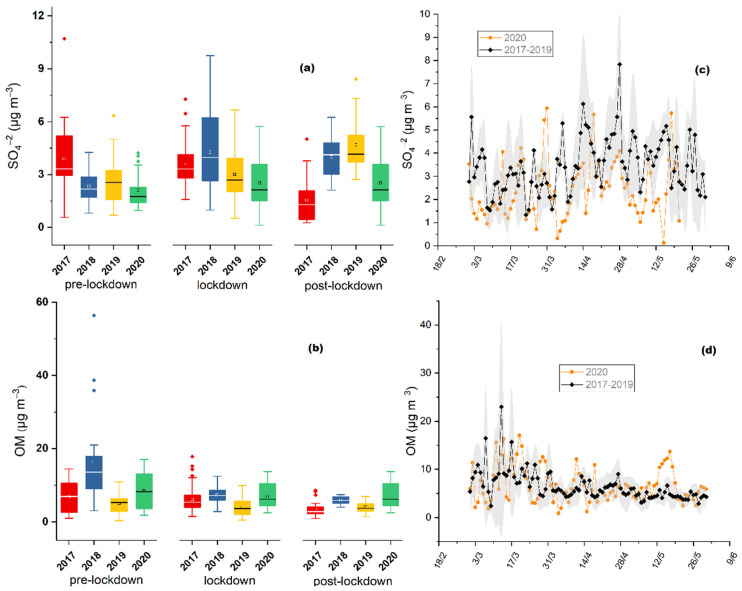
Average values and standard deviations of (**a**) SO_4_^2−^ concentrations (μg m^−3^) and (**b**) OM concentrations (μg m^−3^); for pre-lockdown (1–22 March), lockdown (23 March–10 May) and post-lockdown period, from 2017 to 2020, at the Thissio site. The dots represent the outlier values; (**c**) SO_4_^2−^ concentrations (μg m^−3^) and (**d**) OM concentrations (μg m^−3^) average of 3-year (2017–2019) daily values (standard deviations marked as grey area), compared to daily values of 2020, from 1 March to 10 May, at the Thissio site.

The daily variability of sulfates during the PreL, LocD and PostL period presents averages of 2.11 ± 1.00, 2.57 ± 1.26 and 2.51 ± 1.60 μg m^−3^ during the PreL, LocD and PostL period, respectively (Figure 4a), showing the higher value of increase among the currently studied parameters during the lockdown period (21.9%, *p* = 0.12, compared to the pre-lockdown period). Grivas et al. [34] demonstrated that, during the lockdown period at our site, there was a significant reduction in traffic emissions associated with a simultaneous decrease in species such as CO and NO_2_, and BC_ff_ (black carbon attributed to fossil fuel emissions). The applied Mann–Whitney test reveals that the differences in sulfate concentrations between the studied periods are not significant, while there are limited reported investigations of sulfuric oxide concentrations, demonstrating either decrease or increase [51,52] during the lockdown conditions, depending on the type of the studied site. In China, Chen et al. [53] reported that sulphate barely decreased during the lockdown period, inhibiting a reduction in PM_2.5_ concentrations. Several urban sites worldwide indicated a reduction in NO_x_ combined with stable/slightly increased levels of SO_2_ and O_3_ [52,53], favoring the formation of SO_4_^2−^ during the lockdown period. The increase in sulfate ambient concentrations, compared to other gaseous primary pollutants at the studied site, could be mainly attributed to operations that remained active such as manufacturing facilities and the augmented power demand of households during the quarantine. Furthermore, the production of sulfates is favored by regional aerosol transport. Therefore, the increase in sulfates could partly explain the increase also observed in OP during the lockdown period, while a future study could include more toxic components of aerosols such as metals, polycyclic aromatic hydrocarbons and quinones.

The daily concentrations of organic matter (OM) (Figure 4d) averaged at 8.50 ± 5.00, 5.85 ± 2.73 and 6.93 ± 3.39 μg m^−3^ during the PreL, LocD and PostL period, respectively (Figure 4c). Furthermore, this parameter presented a decrease during the lockdown period (31%, *p* = 0.08, and 16%, *p* = 0.29, compared to the pre- and post-lockdown period, respectively), indicating that there was a statistically significant difference solely concerning the pre-lockdown period. During the LocD period, there is a combined decrease in OM and an increase in sulfates—when compared to the PreL—that could possibly interpret the absence of significant changes in the levels of OP. In regards to BC concentrations, Grivas et al. [36] investigated the BC_ff_ and BC_bb_ components during the same three studied periods (PreL, LocD and PostL); revealing that the component that is mostly related to fossil fuel combustion, BC_ff_, decreased during the LocD period, similarly to the rest of the traffic-related emissions by about 30%. While the BC_bb_ fraction, which is mostly attributed to biomass burning for domestic heating purposes, presented a regularly observed decrease during the days of the LocD period, since the rising temperatures resulted in a reduction in residential heating activities.

### 3.4. Changes in OP, OM and Sulfates between 2020 and Years 2017–2019

To study the yearly variability of aerosol OP, PM_2.5_, OM and SO_4_^2−^ concentrations, the 3-year average values of the studied parameters were calculated for the years 2017–2019 and were compared to those observed during 2020. The aim was to verify whether the observed variation of the studied parameters could be attributed to the lockdown conditions, rather than to repeated seasonal patterns.

During the lockdown period (LocD) the levels of OP (Figure 2a) were reduced by 30% (*p* < 0.001), while those of PM_2.5_, OM and SO_4_^2−^ were lower by 21% (*p* < 0.05), 3% (*p* = 0.52) and 29% (*p* < 0.001), respectively, compared to the average value of the three previous years. These changes were accompanied by a decreasing tendency during the pre- and post- lockdown periods of 2020, as well. More specifically, in the PreL period OP, PM_2.5_, OM and SO_4_^2−^ were lower by 18% (*p* = 0.34), 35 % (*p* < 0.05), 10% (*p* = 0.49) and 30% (*p* < 0.01), respectively, compared to the 3-year average values; while the corresponding reductions in the PostL period of 2020 was 37% (*p* < 0.05), 14% (*p* = 0.1) and 29 % (*p* < 0.05); with the exception of OM which presented an increase of 54% (*p* = 0.01) in 2020, compared to the previous 3 years. The comparative evaluation showed that the observed reductions are not exclusively linked to the lockdown limitations, since the studied parameters reveal the same behavior among all three examined periods of 2020. Other studies that performed similar comparisons between COVID-19 lockdown periods and the previous years (up to 10 previous years), reported a reduction for PM_2.5_ mass concentrations from 19% to 54%, during 2020 ([8] and references therein).

Figure 2c, Figure 3b, and Figure 4c,d demonstrate the obtained time-series of OP, PM_2.5_, sulfate and OM, respectively, for the PreL, LocD and PostL period of 2020 comparing it with the respective data of the same time periods during the years 2017–2019. The 3-year average values and corresponding standard deviations are shown, in order to assess the seasonal variations profile of the investigated aerosol parameters. For all examined time periods (PreL, LocD, PostL) the aerosol OP, PM_2.5_, OM and SO_4_^2−^ concentrations presented levels within the range of the previous years. During the lockdown period of 2020 in Athens, Grivas et al. [35] demonstrated an overall decline in NO_2_ and BC_ff_, in comparison with the previous years; while BC_bb_ appeared to remain stable, following the pattern of aerosol OP. Additionally, even though all traffic related emissions have been significantly reduced [35], there is no depletion in water-soluble aerosol OP. Paraskevopoulou et al. [22] indicated the existence of two major aerosol OP sources in Athens. During winter-time, primary emissions from wood burning for domestic heating purposes are the dominant source of water-soluble OP; whereas, during the warmest period of the year, secondary long-range transported aerosol appears to contribute significantly to aerosol toxicity. Despite the cutbacks of most of the emissions during the lockdown, the almost stable BC_bb_ and sulfate levels mainly driven by primary biomass burning and secondary long-range particulate matter processes, respectively, could explain the stability in OP levels during the lockdown period not only throughout the years but also between pre and post-lock down periods. Moreover, the current unique aerosol data base reveals, for the first time, that traffic-related species conduce to a minimum degree the induction of aerosol reactive oxygen species.

## 4. Conclusions

The current study evaluates the results on aerosol OP upon reduced air pollution emissions in Athens, during the lockdown implemented in 2020. Measurements of PM_2.5_ chemical components and subsequent identification of reactive oxygen species are scarce during the lockdown periods. This case study attempts to shed further light on the aerosol sources and components linked to atmospheric particles’ toxicity. According to the observed PM_2.5_ mass concentration and chemical speciation, long-range transport patterns of aerosol masses, along with local combustion PM sources appear to be significant contributors of the water-soluble OP.

The following insights obtained from our study are listed below:

- During the lockdown period, the levels of aerosol OP were not significantly modified (compared to the pre- and post- lockdown period), presenting a similar pattern with SO_4_^2−^ concentration that is representative of long-range aerosol secondary processes.
- The PM_2.5_ mass showed a small dependence on the cutbacks of emissions, highlighting its correlation with atmospheric processing such as secondary aerosol formation.
- While the levels of NO_2_ and BC_ff_ decreased during the lockdown period of 2020, BC_bb_ values in 2020 displayed similar levels as in the previous years. Aerosol OP presented levels within the range of the previous years, highlighting its correlation with (BC_bb_) biomass burning processes
- The aerosol components that were reduced during the lockdown period do not seem to affect the levels of PM-induced OP. On the contrary, OP appears to be mostly attributed to the local source of biomass burning and regional transportation linked to SO_4_^2−^ concentrations.
- Reduced vehicular emissions, appear to have a minor influence on the aerosol oxidative potential.

Overall, vehicular emissions were reduced around 30%, while previous studies have indicated that traffic contributes roughly 20% of the total aerosol mass [32]. As a consequence, the observed minimal decrease in PM_2.5_ mass (6–7%) is expectedly well in agreement with the reported observations. On the other hand, main contributors of OP, namely biomass burning products and SO_4_^2−^, did not show any significant change. Thus, the decrease in traffic by 30% was not sufficient to drive any measurable change in OP, which, for our site, is found mainly to be driven by wood combustion and regional particulate matter.

Finally, it should be taken into consideration that during the lockdown period, despite the small decrease in PM_2.5_ mass concentrations, the aerosol OP exhibited a positive shift, further amplifying the conclusion that the aerosol chemical species (rather than the aerosol mass concentration) is the most important factor of aerosol toxicity. This highlights the necessity to further study and comprehend the major sources of OP depending on the dominance of aerosol emissions.

## Data Availability

Data are available upon request to the corresponding authors.
